# SARS-CoV-2 in the Amazon region: A harbinger of doom for Amerindians

**DOI:** 10.1371/journal.pntd.0008686

**Published:** 2020-10-29

**Authors:** Juan David Ramírez, Emilia Mia Sordillo, Eduardo Gotuzzo, Carol Zavaleta, Daniel Caplivski, Juan Carlos Navarro, James Lee Crainey, Sergio Luiz Bessa Luz, Lourdes A. Delgado Noguera, Roxane Schaub, Cyril Rousseau, Giovanny Herrera, Maria A Oliveira-Miranda, Maria Teresa Quispe-Vargas, Peter J. Hotez, Alberto Paniz Mondolfi

**Affiliations:** 1 Grupo de Investigaciones Microbiológicas – UR (GIMUR), Departamento de Biología, Facultad de Ciencias Naturales, Universidad del Rosario. Bogotá, Colombia; 2 Icahn School of Medicine at Mount Sinai, New York, United States; 3 Universidad Peruana Cayetano Heredia. Lima, Peru; 4 Universidad Internacional SEK, Quito, Ecuador; 5 Instituto Leônidas e Maria Deane/ILMD/FIOCRUZ, Manaus, Brasil; 6 Instituto de Investigaciones Biomedicas IDB / Emerging Pathogens Network-Incubadora Venezolana de la Ciencia, Cabudare, Venezuela; 7 CIC AG/Inserm 1424, Centre Hospitalier de Cayenne Andrée Rosemon, Cayenne, French Guiana; 8 Laboratoire des Ecosystèmes Amazoniens et Pathologie Tropicale (EPaT) EA 3593, Université de Guyane, Labex CEBA, DFR Santé, Cayenne, French Guiana; 9 Santé publique France. French National Public Health Agency. Regional unit. French Guiana. France; 10 Wataniba Amazon Social Workgroup, Caracas, Venezuela; 11 National School of Tropical Medicine, Baylor College of Medicine, Houston, United States; 12 Academia Nacional de Medicina de Venezuela, Caracas, Venezuela; National Research Centre, EGYPT

## Abstract

As the Severe Acute Respiratory Syndrome Coronavirus 2 (SARS-CoV-2) pandemic continues to expand, healthcare resources globally have been spread thin. Now, the disease is rapidly spreading across South America, with deadly consequences in areas with already weakened public health systems. The Amazon region is particularly susceptible to the widespread devastation from Coronavirus disease 2019 (COVID-19) because of its immunologically fragile native Amerindian inhabitants and epidemiologic vulnerabilities. Herein, we discuss the current situation and potential impact of COVID-19 in the Amazon region and how further spread of the epidemic wave could prove devastating for many Amerindian people living in the Amazon rainforest.

## Introduction

As the Severe Acute Respiratory Syndrome Coronavirus 2 (SARS-CoV-2) pandemic continues to expand, healthcare resources globally have been spread thin. Now, the disease is rapidly spreading across South America, with deadly consequences in areas with already weakened public health systems. [1]. The Amazon region is particularly susceptible to the widespread devastation from Coronavirus disease 2019 (COVID-19), because of its immunologically fragile native Amerindian inhabitants, epidemiologic vulnerabilities due to remoteness, lack of infrastructure, habitat destruction from deforestation and mining, and, ever-present risk for introduction and emergence of new pathogens. [2–3] For the Indigenous peoples of the Amazon, whose numbers have been declining, any death represents a threat to the survival of the tribe.

The introduction of novel infectious diseases may be the most devastating consequence of European colonization into the Americas, particularly in native Indigenous communities, [2] commonly resulting in severe clinical outcomes, increased mortality, and social unrest. The small Indigenous communities in Amazonia (defined as the Amazon River basin and Guiana Shield), have been especially vulnerable to these “virgin soil epidemics,” not only at the time of initial European colonization but during subsequent encounters with settlers and incursions by global industrial interests. [2] The particular vulnerability of New World Indigenous populations, especially Amazonians, is thought due to the high genetic homozygosity that has been proposed to be the consequence of a serial founder effect, compounded by successive generations of inbreeding. [2] The lack of genetic diversity has been shown to promote devastating epidemics of genetically similar hosts for pathogens including malaria, tuberculosis, HIV, and leprosy. [2] In addition to their biological vulnerability, Indigenous groups are further affected by extensive rooted socioeconomic disadvantages, which continue to accelerate in a uniform pattern across countries, translating into the worst health and developmental indicators within nations. [4] In this setting, the reemergence of measles in Venezuela and Brazil has had dire consequences with large numbers of deaths among Yanomami and other Indigenous populations, [5–6] and, now, the SARS-CoV-2 pandemic has the potential to become an extinction event for many of the remaining tribes.

The number of SARS-CoV-2 infections have increased rapidly throughout South America, following the diagnosis of the first COVID-19 case on February 25, 2020, in Sao Paolo, Brazil, and the first reported COVID-19 death, on March 7, 2020, in Argentina. Despite implementation of containment measures, including quarantine and lockdown by several countries, by March 20, 2020, COVID-19 cases had been reported by all South American countries, and according to the World Health Organization, by July 23, 2020, there had been more than 3,300,000 SARS-CoV-2 infections and 121,000 deaths. [7] The 9 countries (Bolivia, Brazil, Colombia, Ecuador, Guiana, French Guiana, Peru, Surinam, and Venezuela) of the Pan-Amazonian region accounted for most of these cases and deaths, led by Brazil (2,227,514 cases and 82,771 deaths), [8] Ecuador (78,148 cases and 5,439 deaths), [9] and Colombia (226,373 cases and more than 7,680 deaths). [10] Although currently there is insufficient data to make confident conclusions regarding the influence of climatic factors such as temperature and humidity on transmission of SARS-CoV-2 [1], we discuss several characteristics for each of the diverse environments within the Amazon region that may enhance transmission.

## Brazil: The example of Amazonas

**Fig 1:**
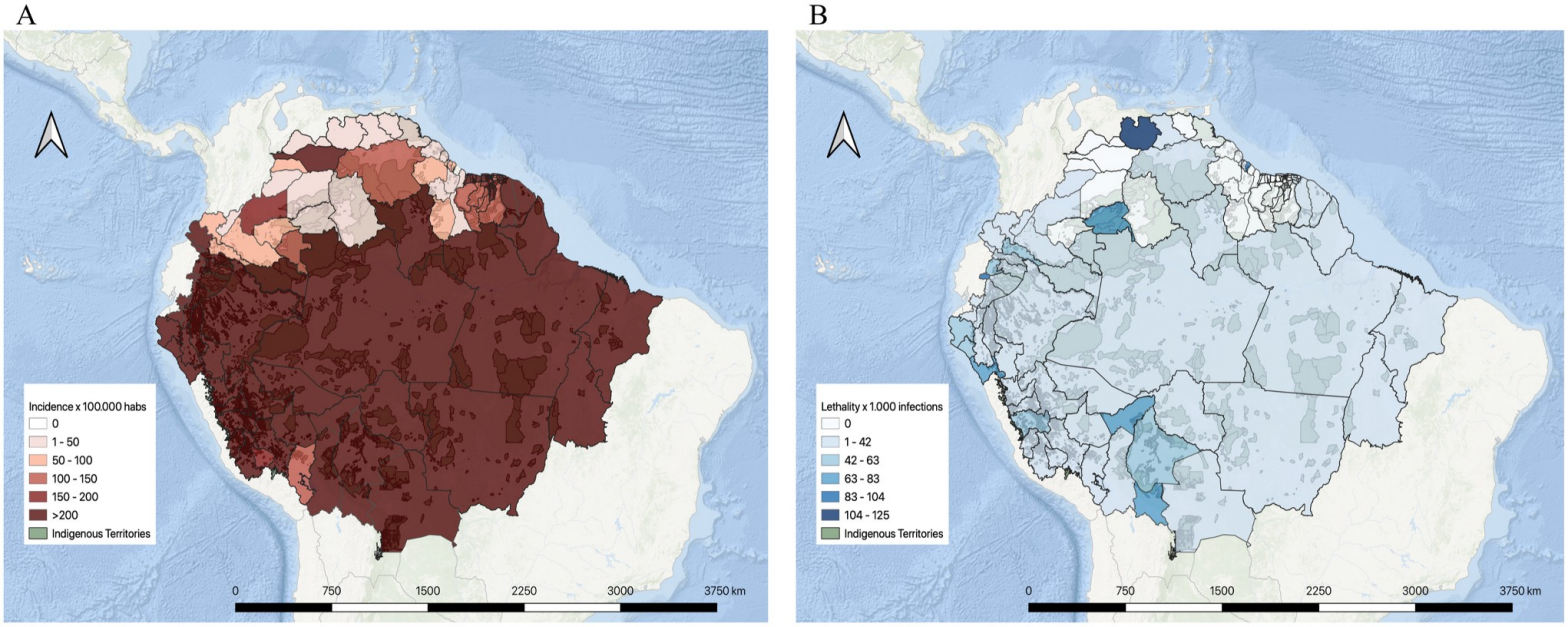
COVID-19 in the Amazonia: infected and deceased. (A) Incidence [29] in cases per 100,000 people is shown in a spectrum of white (zero incidence) to dark red (more than 200 cases per 100,000). (B) Lethality [29] rates per 1,000 infections is shown in a spectrum of white (zero deaths) to dark blue (104 to 125 deaths). The Indigenous territories are indicated in light green. [30] The map was created with the QGIS software (https://www.qgis.org/es/site/). [31]

Within Brazil, the state of Amazonas has felt the greatest impact thus far. Amazonas is the Brazilian state with the largest number of native Indigenous people, from the more populous tribes of the Tikuna and Yanomami people as well as from smaller tribes, some of which, like the Akuntsu people, have only a few remaining members. In Manaus, the capital city of Amazonas, the convergence of a particularly vulnerable population, a new pathogen and limited healthcare facilities and resources created a “perfect storm,” evidenced by the dramatic rise in confirmed SARS-CoV-2 infections, which jumped from 67 to 32,496 cases (more than 1,000%) between March 26 and July 23 (Fig 1A; Table 1) [11–12]. In Manaus, there are 293 hospital beds (private and public) and 8 ambulances, to serve its approximately 2 million inhabitants. According to the State Health Secretariat, all intensive care unit (ICU) beds were occupied on May 1; there are no available ICU beds in hospitals outside Manaus [13]. The lethality rate in Manaus is the highest in Brazil (6.07% by July 23; Fig 1B), necessitating the use of mass graves to accommodate an average of 180 burials daily. [12]

**Table 1:** Summary of COVID-19 cases in the Amazon region by state or department.

Country	Amazon State or Department^30^	Confirmed cases^29^	Deaths^29^
Bolivia	Cochabamba	6,277	468
	Beni	5,068	281
	La Paz	8,922	157
	Pando	1,178	94
	Santa Cruz	29,486	797
Brazil	Acre	17,295	460
	Roraima	25,467	429
	Amapá	33,705	507
	Mato Grosso	34,136	1,332
	Rondônia	29,117	686
	Amazonas	90,534	3,129
	Maranhão	106,092	2,676
	Pará	138,396	5,538
	Tocantins	17,773	294
Colombia	Caquetá	220	4
	Amazonas	2,454	101
	Guaviare	48	0
	Putumayo	199	11
	Vaupés	61	1
	Casanare	148	1
	Guainía	14	1
	Arauca	161	0
	Huila	520	15
	Meta	1,895	20
	Vichada	2	0
	Nariño	5,194	163
	Cauca	1,055	34
Ecuador	Pastaza	1,119	36
	Zamora Chinchipe	792	28
	Tungurahua	1,700	157
	Sucumbíos	822	25
	Orellana	1,073	30
	Morona Santiago	1,590	13
	Napo	814	48
	Azuay	2,651	70
	Carchi	587	20
	Loja	1,712	59
French Guiana	Saint-Laurent-du-Maroni	588	12
	Cayenne	6,066	25
Guyana	Cuyuni-Mazaruni	11	0
	Pomeroon-Supenaam	3	0
	East Berbice-Corentyne	11	0
	Barima-Waini	11	0
	Upper Takutu-Upper Essequibo	11	0
	Upper Demerara-Berbice	11	0
	Essequibo Islands-West Demerara	97	4
	Potaro-Siparuni	4	0
	Mahaica-Berbice	0	0
	Demerara-Mahaica	179	15
Peru	Apurimac	715	27
	Madre De Dios	2,605	83
	Cusco	2,750	41
	Loreto	10,227	369
	Pasco	1,454	37
	Huanuco	4,836	134
	San Martin	7,019	194
	Amazonas	3,994	98
	Ucayali	9,157	177
	Ayacucho	2,791	43
	Cajamarca	4,205	144
	Piura	19,463	874
	Lambayeque	15,753	819
	Huancavelica	1,236	27
	La Libertad	13,074	876
	Junin	5,875	285
	Puno	1,299	49
Suriname	Para	40	0
	Saramacca	40	0
	Marowijne	220	9
	Sipaliwini	50	0
	Wanica	130	0
	Commewijne	40	0
	Nickerie	100	0
	Paramaribo	301	12
	Coronie	40	0
	Brokopondo	40	0
Venezuela	Amazonas	35	0
	Bolivar	995	5
	Delta Amacuro	30	0

Despite a current downward trend in hospitalizations, Manaus continues to have the most confirmed SARS-CoV-2 cases, followed by other Amazonian municipalities such as Coari, Manacapuru, and São Gabriel da Cachoeira [12]. The convergence of urban and suburban overcrowding, poor water and sanitation systems and delayed government response are factors contributing to the recent upsurge in cases. In addition, the close contact between families and communities in Amerindian settings, or during riverboat transportation, further fuels the epidemiological dynamics of COVID-19.

## Colombia

A similarly disastrous situation is occurring in the Amazonas department of Colombia. Leticia, the capital of the Amazonas department, which is a city of 80,000 inhabitants (the majority of whom are Indigenous peoples), reported its first COVID-19 case on April 17. By July 23, 2020, the number of cases had jumped to 2,335 cases with 98 deaths, representing the highest number of COVID-19 cases per capita in Colombia (Fig 1A) [10]. The Colombian Amazon region, as in Brazil, is home to a large Indigenous population, potentially highly vulnerable to rapid expansion of the virus. Among the 54 Indigenous ethnic groups, approximately 398,365 Indigenous families are in risk, most confirmed SARS-CoV-2 infections have been in the Zenú, Tikuna, Mokaná, Uitoto, Los Pastos, and Pijao ethnic groups; the several deaths pose a risk for these shrinking communities [14]. Leticia and its populace are also at risk for directional spread from the border city of Tabatinga, Brazil, which has had about 1,629 COVID-19 cases and 78 deaths to date (Fig 1A and 1B; Table 1) [12]. Here again, resources to fight this pandemic are severely limited. There are only 2 hospitals in Leticia city, totaling 70 beds. Only 1 of the 2 has an ICU, with 23 beds, all currently occupied [15].

## Ecuador

The 6 provinces (Sucumbíos, Orellana, Napo, Pastaza, Morona Santiago, and Zamora Chinchipe) in the Amazon region of Ecuador have the lowest population density in the country, with 12% of the total population but with only 10 ICUs and 19 critical care beds [16]. Nine Indigenous ethnic groups are registered in the region, including 7 crossborder, and 2 uncontacted or voluntarily isolated ethnic groups. Colonists (mestizos) and the Kichwa ethnic group who have migrated from the highlands since the 1950s, are the largest part of the population, outnumbering the ethnic groups that originally inhabited the area. The first apparent case of COVID-19 in Ecuador was reported on February 29, in a traveler returning from Spain, and the first case in Ecuador’s Amazon region on March 7, in a Dutch tourist [17]. By July 23, 2020, there were 78,148 cases registered in Ecuador, mostly in the Costa and the Sierra, although this likely reflects underreporting due to limited resources, for diagnosis and testing (Fig 1A; Table 1) [9]. Official statistics do not differentiate cases between settlers and Indigenous ethnic group; cases affecting Indigenous people are mostly based on clinical findings, with few confirmed by rapid tests. In late April and early May, 2 deaths and 14 COVID-19 cases confirmed by rapid tests were also reported in the Secoya (Siekopai) community in Sucumbios, a crossborder (Peru) community with fewer than 1,000 inhabitants [18]. As of mid-July, 699 cases have been reported among Kichwas in the province of Napo, the highest number thus far among Indigenous ethnic groups [19]. Besides, the Waorani community, one of the most homozygous and with only about 2,000 members, reported 297 cases by mid-July, and, also, 22 cases were reported among the Achuar in the Pastaza province [19]. Scarcity of resources severely hampers efforts to contain the epidemic and limit spread to other vulnerable ethnic groups in the region.

## Peru

The COVID-19 catastrophe in the Peruvian Amazon has already begun in Iquitos, the capital of the Loreto region. Loreto is home of the greatest number of Amazon Indigenous communities in Peru. The lack of basic health resources, such as supplemental oxygen, has been a major limitation for treatment of SARS-CoV-2 infected patients, even among those who do not require intubation. As a consequence, Loreto has one of the highest estimated case fatality rates in Peru, 5.1% compared to 4.7% in the rest of the country (Fig 1B) [20]. Although reports of cases nation-wide have not included ethnicity, a few regions have begun to include this information in order to better understand the impact of the COVID-19 epidemic on Indigenous people. A report from the Ucayali Amazon region indicated that of a total of 3,525 SARS-CoV-2 positive cases, 41 were members of the Shipibo Indigenous people [21]. In the Alto Amazonas Province, a report from the Catholic church indicated that of a total of 272 confirmed cases, 3 patients were Kukama and 2 were Shawi people [22].

According to the last Peruvian National census, only 865 (32%) of a total of 2,703 native communities had a health post, and virtually none had facilities to admit patients [23]. Therefore, the urgent need to improve health resources in the midst of the COVID-19 pandemic represents one of the greatest challenges to protect the survival of Indigenous people. Although some Indigenous people may be able to self-isolate in the forest, natural resource degradation affecting food and water quality in Peruvian Amazon [24] and the increasing dependency of Indigenous households on the market to access cash and food supplies have made mobility and self-isolation more difficult [25]. Protection of the natural environment and provision of food aid are of primary importance to enhance the viability of self-isolation as a strategy for Indigenous communities.

## French Guiana

The onset of the COVID-19 epidemic in French Guiana, a French overseas department located between Suriname and the Brazilian state of Amapá, lagged its onset in mainland France by several weeks. The early implementation by the French government of a strict lockdown, simultaneously in mainland France and French Guiana, enabled containment of the epidemic at an early stage. Hence, 6,654 cases and 37 deaths had been registered as of mid-July (Fig 1A and 1B; Table 1) [26]. Many of the earliest cases reported were in travelers from mainland France and their contacts, often in small intrafamilial clusters. One large community cluster reported at the beginning of the epidemic occurred in an Amerindian community located close to the capital city, in which 21 cases were detected in an extended family group of 60 people, living in a village of approximately 250 inhabitants [26]. The chains of transmission in that community were stopped by active early screening and rapid isolation of the village.

Despite the early lockdown and apparent containment, a recent surge in cases has been observed, following lifting of the lockdown on May 11. Notably, clusters have arisen in several Amerindian communities on the Oyapock river, in relation with cases imported from bordering Brazil [26]. This surge may have been favored by the difficulties of food insecurity, forcing the community to seek supplies in Brazil, and exacerbated by a sociocultural context that mixes precariousness, frequent contacts with the extended family, and, sometimes, low cultural acceptance of lockdown measures. Furthermore, the remote Amerindian communities on the upper Oyapock river and the upper Maroni river bordering Suriname are at increased risk due to the movement of illegal gold miners whose activity has intensified since the onset of the epidemic.

## Venezuela

Even before the COVID-19 pandemic, Venezuela had suffered a massive resurgence in both vaccine-preventable infections, especially measles, and neglected tropical diseases, due to extreme socioeconomic instability and lapses in public health control measures. Now, strict censoring of epidemiological data has limited the availability of direct information about the course and impact of the COVID-19 epidemic in Venezuela. However, the highly mobile population and permeable borders between Venezuela and its neighbors, Colombia and Brazil, act as a disease corridor between countries and provide a window into the status of the outbreak in Venezuela [27]. Mobilization through the borders has increased dramatically in conjunction with the widely reported increase in illegal mining activities in the Venezuelan Amazon. COVID-19 cases have already been reported in the 3 states of the Guyana shield region, first in the Bolivar State, specifically in the municipalities of Gran Sabana and now reported in the Heres and Caroní municipalities. Currently, in the Amazon State, there have been 36 cases reported and 33 in the Delta Amacuro State; none are recognized as Indigenous (Fig 1A; Table 1) [28]. Although the Venezuelan government usually does not report cases differentiated by Indigenous or nonindigenous peoples or Indigenous tribes, it is known that 146 of the diagnosed cases are Indigenous, occurring in 6 Yeral, 3 Kurripakos, 127 Pemon, 4 Warao, and 6 without ethnic identification, all of whom are associated with Brazilian cases mainly from São Gabriel de Cachoeira municipality [29]. They were immediately put into quarantine and have been receiving medical assistance. Nevertheless, travel through the Venezuelan–Brazilian border and the Venezuelan–Colombian border through illegal paths is known to be an ongoing concern that has not been addressed by the government. Furthermore, some COVID-19 cases have been diagnosed in the Yanomami and Warao people who reside across the Brazilian–Venezuelan border; the Wayuu Indigenous people on the Colombia–Venezuelan border are similarly vulnerable [29].

Facing the worst humanitarian refugee crisis ever witnessed in the Western hemisphere and a total breakdown of its healthcare and public health systems, Venezuela will not only be unable to face the challenges of this ongoing pandemic but, also, could dangerously serve as a disease-amplifier, also putting at risk the already vulnerable healthcare systems of its neighbors.

## A call for action

Although many countries and regions around the world have implemented lockdown policies to halt viral transmission, such an approach may prove ineffective given the unique geographic idiosyncrasy of the Amazon. Most areas of the vast rainforest rely on river transportation for commerce and as the main form of regional transport. With myriad labyrinthine waterways, lockdown measures to contain population movement are nearly impossible.

Measures to address the imminent effects of the arrival of SARS-CoV-2 in the Amazon region and the potentially devastating impact on the most vulnerable Amerindian aboriginal settlements must include: intensified epidemiologic surveillance and enhanced reporting to track spread of the virus; mobilization of necessary resources for healthcare delivery; establishment of rapid response systems to ensure food security and resources to the hardest-hit areas; marshaling deployment of specialized field healthcare personnel and all necessary resources to provide onsite testing and critical care to affected patients; and mobilization of necessary security forces to restrict any illegal foreign incursions to Indigenous territories, particularly illegal miners. Regional and global efforts will be required to ensure that these vulnerable populations receive timely access to new COVID-19 vaccines and therapies as they become available.

Although governments must develop nationwide strategies to mitigate the effects of the SARS-CoV2 pandemic, the particular vulnerability of native Indigenous peoples, and potential for loss of entire communities highlights the need for special attention. Recently, the Office of the United Nations High Commissioner for Human Rights issued a statement declaring that “Indigenous people will face extreme risks.” As many Amazonian countries rapidly become epicenters of COVID-19, governments should take timely and decisive actions to tackle this potentially overwhelming ethnic crisis.

Together, the measles epidemic and COVID-19 pandemic represent a “one-two punch” that might accelerate catastrophic declines to the public health of Indigenous populations in the region. Further spread of the latest COVID-19 epidemic wave could prove devastating for many Amerindian people living in the Amazon rainforest, ultimately pushing these communities towards extinction.
