# Correction: SARS-CoV-2 in the Amazon region: A harbinger of doom for Amerindians

**DOI:** 10.1371/journal.pntd.0009118

**Published:** 2021-02-09

**Authors:** Juan David Ramírez, Emilia Mia Sordillo, Eduardo Gotuzzo, Carol Zavaleta, Daniel Caplivski, Juan Carlos Navarro, James Lee Crainey, Sergio Luiz Bessa Luz, Lourdes A. Delgado Noguera, Roxane Schaub, Cyril Rousseau, Giovanny Herrera, Maria A Oliveira-Miranda, Maria Teresa Quispe-Vargas, Peter J. Hotez, Alberto Paniz Mondolfi

There is an error in the article XML causing the corresponding author’s initials to be indexed incorrectly. The correct initials are: Paniz Mondolfi A.

The correct citation is: Ramírez JD, Sordillo EM, Gotuzzo E, Zavaleta C, Caplivski D, Navarro JC, et al. (2020) SARS-CoV-2 in the Amazon region: A harbinger of doom for Amerindians. PLoS Negl Trop Dis 14(10): e0008686. https://doi.org/10.1371/journal.pntd.0008686
